# Inter-organellar Communication in Parkinson's and Alzheimer's Disease: Looking Beyond Endoplasmic Reticulum-Mitochondria Contact Sites

**DOI:** 10.3389/fnins.2022.900338

**Published:** 2022-06-21

**Authors:** Stephanie Vrijsen, Céline Vrancx, Mara Del Vecchio, Johannes V. Swinnen, Patrizia Agostinis, Joris Winderickx, Peter Vangheluwe, Wim Annaert

**Affiliations:** ^1^Laboratory of Cellular Transport Systems, Department of Cellular and Molecular Medicine, Katholieke Universiteit Leuven (KU Leuven), Leuven, Belgium; ^2^Aligning Science Across Parkinson's (ASAP) Collaborative Research Network, KU Leuven, Leuven, Belgium; ^3^Laboratory for Membrane Trafficking, VIB-Center for Brain and Disease Research, KU Leuven, Leuven, Belgium; ^4^Department of Neurosciences, KU Leuven, Leuven, Belgium; ^5^Laboratory of Functional Biology, Department of Biology, KU Leuven, Heverlee, Belgium; ^6^Laboratory of Lipid Metabolism and Cancer, Department of Oncology, Leuven Cancer Institute (LKI), KU Leuven, Leuven, Belgium; ^7^Laboratory of Cell Death Research and Therapy, VIB-Center for Cancer Research, KU Leuven, Leuven, Belgium; ^8^Department of Cellular and Molecular Medicine, KU Leuven, Leuven, Belgium

**Keywords:** endolysosome, mitochondria, neurodegenerative disease, inter-organellar communication, lipid metabolism, membrane contact site

## Abstract

Neurodegenerative diseases (NDs) are generally considered proteinopathies but whereas this may initiate disease in familial cases, onset in sporadic diseases may originate from a gradually disrupted organellar homeostasis. Herein, endolysosomal abnormalities, mitochondrial dysfunction, endoplasmic reticulum (ER) stress, and altered lipid metabolism are commonly observed in early preclinical stages of major NDs, including Parkinson's disease (PD) and Alzheimer's disease (AD). Among the multitude of underlying defective molecular mechanisms that have been suggested in the past decades, dysregulation of inter-organellar communication through the so-called membrane contact sites (MCSs) is becoming increasingly apparent. Although MCSs exist between almost every other type of subcellular organelle, to date, most focus has been put on defective communication between the ER and mitochondria in NDs, given these compartments are critical in neuronal survival. Contributions of other MCSs, notably those with endolysosomes and lipid droplets are emerging, supported as well by genetic studies, identifying genes functionally involved in lysosomal homeostasis. In this review, we summarize the molecular identity of the organelle interactome in yeast and mammalian cells, and critically evaluate the evidence supporting the contribution of disturbed MCSs to the general disrupted inter-organellar homeostasis in NDs, taking PD and AD as major examples.

## Introduction

Eukaryotic cells rely on their organelles to maintain cellular functions and processes, which are crucial in governing the efficacy of cellular responses to environmental and developmental changes or pathological signals. An efficient coordination and communication between organelles is of utmost importance to warrant cell survival, especially in the case of neurons. For a long time, it was thought that the inter-organellar exchange of signals and metabolites occurred primarily through vesicular trafficking as well as metabolic exchange and enzyme translocations *via* diffusion across the cytoplasm. This view has fundamentally changed during the past decades with the observation that every organelle type forms functional contacts with at least one other type. At these so-called membrane contact sites (MCSs), the membranes of distinct organelles do not fuse, but are physically connected through proteinaceous tethers, thereby establishing a platform for the exchange of lipids, ions and metabolites (Bohnert, 2020; Huang et al., 2020). Interestingly, MCSs have also been described between organelles and lipid droplets (LDs) (Renne and Hariri, 2021; Rakotonirina-Ricquebourg et al., 2022) and even within organelles, like the contact that is formed between the inner and outer membranes of mitochondria, which is known as MICOS (mitochondrial contact site and cristae organizing system) and is involved in establishing the architecture of the respiratory chain, lipid metabolism, and protein import into mitochondria (Kozjak-Pavlovic, 2017; Eramo et al., 2020). Over the past years, also the field of neurodegeneration research is becoming increasingly aware of the importance of inter-organellar communication in neuronal survival. Given that mitochondrial defects are a common feature to most neurodegenerative diseases (NDs), including Parkinson's (PD) and Alzheimer's (AD), most attention has been given to contacts involving mitochondria, specifically with the endoplasmic reticulum (ER) [for a detailed review, see (Gómez-Suaga et al., 2018; Raeisossadati and Ferrari, 2020; Leal and Martins, 2021; Lim et al., 2021; Ray et al., 2021; Ziegler et al., 2021)]. However, more recent observations fail to reconcile different NDs-associated cellular mechanisms such as endolysosomal disruption, with sole impairments in ER-mitochondria contact sites (Petkovic et al., 2021). Therefore, the global objective of this review is to broaden the scope of inter-organellar contacts in NDs. We will describe the main MCSs related to PD and AD-associated cellular impairments, thereby not only focusing on their molecular determinants and structural properties but also on their functional implications and interplay with one another. Importantly, we will link the current knowledge to the available information of MCSs interactor orthologs identified in yeast, in order to propose putative tethers and mechanisms involving mammalian MCSs that have been overlooked until now. Together, this review aims to provide a conceptual framework for future endeavors aiming at elucidating how the organelle interactome is impaired in and/or contributes to neurodegeneration.

## Inter-Organellar Membrane Contact Sites Composition: Conserved From Yeast to Mammalian Cells?

**Figure 1:**
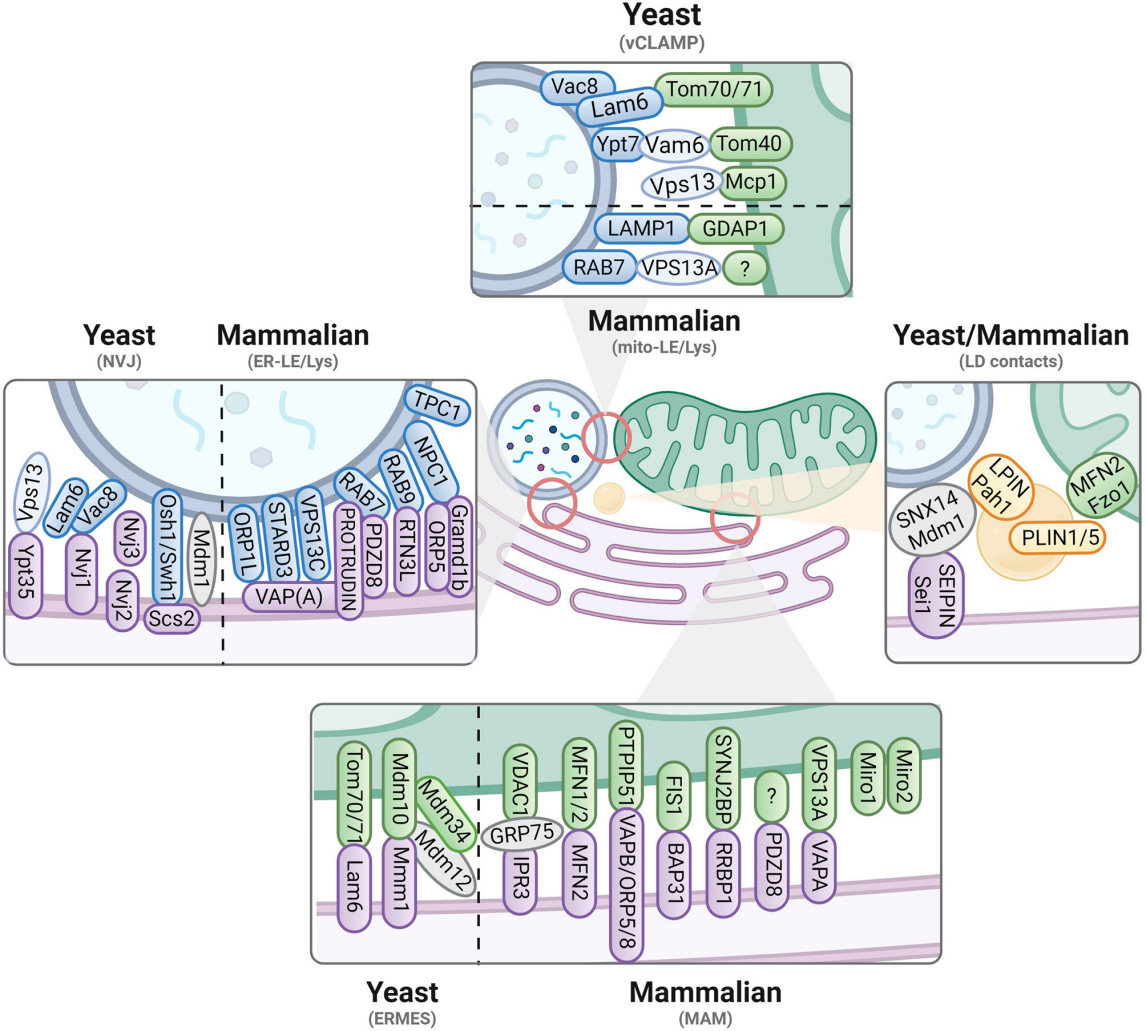
Contact sites in yeast and mammalian cells. Known tethers and interactors are displayed for contacts involving the ER (magenta), mitochondria (green), vacuoles/endolysosomes (blue) and lipid droplets (yellow). Yeast *versus* mammalian interactors are separated where possible. Proteins identified in yeast are written in lowercase, while mammalian/human proteins are in capital letters. For more details, we refer to Table 1.

The understanding that each organelle in a cell can communicate through MCSs with others to ensure particular functions unsurprisingly led to the idea that disruptions in this interactome would contribute to disease outcome, *i.e.*, neuronal death in the case of NDs. To elucidate the cellular mechanisms at stake, the identification of molecular players at MCSs is therefore crucial. Important lessons can be taken from the discoveries gathered in yeast models, which have been extensively characterized over the past years. In this section, we will detail the structural properties and known tethers at specific inter-organellar contacts in both yeast and mammalian cells (Figure 1; Table 1), underlining their potentially shared functional involvement in cellular homeostasis. Considering that mitochondrial and endolysosomal demise are major hallmarks of cellular impairments in PD and AD, we mostly focus on MCSs involving these compartments.

**Table 1:** Molecular determinants for inter-organellar tethering.

**YEAST**			
***Tethering complexes***	***Mammalian/human homologs***	***Functional property***	***References***
**ERMES**			
Mdm10 - Mdm12 - Mdm34 - Mmm1	n.a. - n.a. - n.a. - PDZD8 (paralog)	All members contain a SMP domain and form a channel-like structure to facilitate phospholipid transport	Kornmann et al., 2009; Jeong et al., 2016
Lam6 - Tom70/71	GRAMD1(A-C)/GRAMD2 - TOMM70	Controls the equilibrium and expansion of contact sites	Elbaz-Alon et al., 2015; Murley et al., 2015
**EMC**			
Emc1/2/3/4/5/6 - Tom5	EMC1/2/3/4/MMGT1/EMC6 - TOMM5	The complex is a transmembrane-domain insertase that sustains phospholipid import into mitochondria	Lahiri et al., 2014
**vCLAMP**			
Ypt7 - Vam6 - Tom40	RAB7 - VPS39 - TOMM40	Ypt7 and Vam6 are core components of HOPS required to maintain viability in the absence of ERMES	Elbaz-Alon et al., 2014; Hönscher et al., 2014; González Montoro et al., 2018
Vps13 - Mcp1	VPS13A/C - n.a.	Vps13 is a lipid transporter that forms a distinct vacuolar-mitochondrial contact required to bypass ERMES	Lang et al., 2015; John Peter et al., 2017; González Montoro et al., 2018; Dziurdzik and Conibear, 2021
Vac8 - Lam6 - Tom70/71	ARVCF - GRAMD1(A-C)/GRAMD2 - TOMM70	Controls the equilibrium and expansion of contact sites	Elbaz-Alon et al., 2015
**ER-vacuole (NVJ)**		LDs biogenesis	
Vac8 - Nvj1	ARVCF - n.a.		Pan et al., 2000; Levine and Munro, 2001; Toulmay and Prinz, 2012; Elbaz-Alon et al., 2015; Henne et al., 2015; Bean et al., 2018; Hariri et al., 2018; Weber-Boyvat et al., 2021
Mdm1	SNX14		Henne et al., 2015; Hariri et al., 2018
Osh1/Swh1 - Scs2	OSBP1/2 - VAPA/B		Levine and Munro, 2001; Weber-Boyvat et al., 2021
Vps13 - Ypt35	VPS13A/C - SNX16		Bean et al., 2018
Vac8 - Lam6	ARVCF - GRAMD1(A-C)/GRAMD2		Elbaz-Alon et al., 2015
Nvj2	TEX2		Toulmay and Prinz, 2012
Nvj3	n.a.		Henne et al., 2015
**Lipid droplet contacts**		Lipid storage and trafficking	
Sei1/Fld1	BSCL2		Fei et al., 2008; Grippa et al., 2015; Hariri et al., 2018
Mdm1	SNX14		Hariri et al., 2018
Pah1	LPIN1/2/3		Barbosa et al., 2015
**MAMMALIAN**			
***Tethering complexes***	***Yeast homologs***	***Functional property***	***References***
**MAM**			
IP_3_R3 - GRP75 - VDAC	n.a. - Ecm10 - Por1/2	Ca^2+^ transfer between ER and mitochondria	Rizzuto et al., 1998; Szabadkai et al., 2006
Mfn2 - Mfn2 or Mfn2 - Mfn1	Fzo1	Ca^2+^ transfer between ER and mitochondria	de Brito and Scorrano, 2008; Cosson et al., 2012
Miro1/2 cluster	Gem1	Controls ER and mitochondria Ca^2+^transfer *via* interaction with IP_3_R3 and VDAC; interacts with MICOS complex	Lee et al., 2016; Modi et al., 2019
BAP31 - FIS1	Yet1/2/3 - Fis1	Involved in transmission of apoptotic signals from mitochondria to ER	Iwasawa et al., 2011
VAPA - VPS13A	Scs2/22 - Vps13	Phospholipid (PA, PS and PE) transfer	Kumar et al., 2018; Muñoz-Braceras et al., 2019; Yeshaw et al., 2019
VAPB - PTPIP51	Scs2/22 - n.a.	Ca^2+^ transfer between ER and mitochondria; regulates autophagy/mitophagy	De Vos et al., 2012; Gomez-Suaga et al., 2017
ORP5/8 - PTPIP51	Osh2/3 - n.a.	Sterol sensing and phospholipid transfer	Galmes et al., 2016; Leal et al., 2020
PDZD8	Mmm1 (paralog)	Ca^2+^ transfer between ER and mitochondria	Hirabayashi et al., 2017
**EMC**			
EMC1/2 - SLC25A46	Emc1/2 - n.a. (Ugo1-like)	Maintenance of mitochondrial phospholipid levels	Janer et al., 2016
**Mito-LE/Lys**			
RAB7 - VPS13A	Ypt7 - Vps13	Required for optimal lysosome degradation capacity	Muñoz-Braceras et al., 2019
GDAP1 - LAMP1	n.a. - n.a.	May regulate the duration and number of mito-LE/Lys contacts	Cantarero et al., 2021
**ER-LE/Lys**		Ca^2+^ and cholesterol transfer between ER and LE/Lys, endolysosomal maturation	
VAPA - ORP1L	Scs2/22 - Osh1-7		Rocha et al., 2009
VAPs - STARD3	Scs2/22 - n.a.		Alpy et al., 2013; Wilhelm et al., 2016; Kumar et al., 2018
VAPs - VPS13C	Scs2/22 - Vps13		Leonzino et al., 2021
Protrudin - RAB7/PI3P - PDZD8	n.a.- Ypt7 - Mmm1		Raiborg et al., 2015; Elbaz-Alon et al., 2020; Khan et al., 2021
RTN3L - RAB9	Rtn1/2 - n.a.		Wu and Voeltz, 2021
ORP5 - NPC1	Osh6 - Ncr1		Du et al., 2011
Gramd1b - NPC1	Lam6 - Ncr1		Höglinger et al., 2019
TPC1	n.a.		Kilpatrick et al., 2017
**Lipid droplet contacts**		Lipid transfer and homeostasis	
Seipin (BSCL2)	Sei1/Fld1		Sui et al., 2018; Benador et al., 2019
Mfn2 - PLIN1/5	Fzo1 - n.a.		Benador et al., 2019
SNX14	Mdm1		Datta et al., 2019

*Known tethers and modulators of MCSs between (i) ER and mitochondria, (ii) mitochondria and vacuole/endolysosome, (iii) ER and vacuole/endolysosome, and (iv) ER-mitochondria-vacuole/endolysosome and lipid droplets are displayed in yeast and mammalian cells respectively. Homologs based on data available in the Saccharomyces Genome Database (www.yeastgenome.org) and Alliance Genome Resources (www.alliancegenome.org) are indicated where applicable. The main functional properties of each contact are summarized to indicate their possible disruption in association with cellular dyshomeostasis. n.a., not available/applicable*.

### Membrane Contact Sites Between the ER and Mitochondria

Although mitochondria were known to contact the ER decades ago (Dalton and Copeland, 1959; Ohmann, 1980; Shiao et al., 1995; Achleitner et al., 1999), insight into the structural basis of these contacts and the tethering proteins involved only emerged in the beginning of this century. In the yeast *Saccharomyces cerevisiae*, these contacts are known as ERMES (ER-mitochondrial encounter structure) and are formed by a complex of four core components, *i.e.*, the outer mitochondrial membrane (OMM) proteins Mdm10 and Mdm34, the bridging protein Mdm12, and the integral ER protein Mmm1 (Kornmann et al., 2009). Mdm34, Mdm12 and Mmm1 share a synaptotagmin-like mitochondrial lipid-binding protein (SMP) domain that directs the assembly of ERMES components into a hydrophobic tunnel-like structure, which was proposed to facilitate the trafficking of phospholipid-like phosphatidic acid (PA) and phosphatidylserine (PS) through the complex (Jeong et al., 2016, 2017). Mdm10, on the other hand, has been linked to ergosterol trafficking (Tan et al., 2013) but also interacts with the sorting and assembly machinery (SAM) complex that mediates the proper assembly of the TOM translocase required for mitochondrial protein import (Meisinger et al., 2004; Yamano et al., 2010b). In addition to these four core components, ERMES involves some peripheral proteins. These include (i) the sterol transporter Lam6, which interacts with the mitochondrial protein import receptor proteins Tom70/71 and acts as positive regulator for ERMES expansion (Elbaz-Alon et al., 2015; Murley et al., 2015), (ii) the TOM translocase modulator Tom7, which promotes the segregation of Mdm10 from the SAM complex thereby enhancing the association of Mdm10 with the Mmm1 complex (Yamano et al., 2010a; Becker et al., 2011), (iii) the calcium (Ca^2+^)-binding Rho-like GTPase Gem1, which plays a modulatory role in controlling the number and size of ERMES (Kornmann et al., 2011; Stroud et al., 2011), and (iv) the ARF family GTPase Sar1, which enhances membrane curvature and acts as a negative regulator of ERMES size (Ackema et al., 2016). More recently, an additional ERMES regulator, named Emr1, was identified in the fission yeast *Schizosaccharomyces pombe*. Emr1 is an OMM protein that physically interacts with Mdm34 and Mdm12 to sustain the formation of ERMES and the proper synthesis of phosphatidylethanolamine (PE) (Rasul et al., 2021). Whether the *S. cerevisiae* homolog Mco6 fulfills a similar function remains to be confirmed.

Albeit most core components of ERMES are absent in metazoans, animal cells do have a functional counterpart, which is known as the mitochondria-associated membranes (MAMs). Similar to ERMES, MAMs serve as hubs for phospholipid and sterol transport and they are crucial for mitochondrial biogenesis. In addition, MAMs also support the transfer of Ca^2+^ from the ER to mitochondria (Becker et al., 1980; Rizzuto et al., 1998; Achleitner et al., 1999; Martin et al., 2016; Szymański et al., 2017). The structure of MAMs is complex as different types of tethers have been proposed to establish these contact sites (Figure 1; Table 1). Known players include tethers formed by (i) the interaction of the ER inositol triphosphate receptor type 3 (IP_3_R3) Ca^2+^ channel with the mitochondrial heat shock protein Hspa9/GRP75 and the voltage dependent anion channel (VDAC) (Rizzuto et al., 1998; Szabadkai et al., 2006), (ii) the dimers formed by the mitofusins Mfn1 and Mfn2 (de Brito and Scorrano, 2008; Cosson et al., 2012), (iii) the clusters formed by the Rho GTPases Miro1 and Miro2 (Lee et al., 2016; Modi et al., 2019), or (iv) the complexes formed between the ER integral transmembrane protein VAPB and the OMM protein PTPIP51 (De Vos et al., 2012; Gomez-Suaga et al., 2017) or between VAPA and the OMM associated lipid transporter family member VPS13A (Kumar et al., 2018; Muñoz-Braceras et al., 2019; Yeshaw et al., 2019). Some of the MAM tethering (*e.g.*, IP_3_R3) and regulatory [*e.g.*, the phosphofurin acidic cluster sortin protein PACS2 (Simmen et al., 2005)] or the PKR-like endoplasmic reticulum kinase PERK (Verfaillie et al., 2012; van Vliet et al., 2017) proteins do not have a yeast homolog, but many of them do (Table 1). However, the functional equivalency for ER-mitochondrial tethering was only determined in more detail for the MAM SMP protein PDZD8 and its yeast paralog Mmm1 (Hirabayashi et al., 2017; Wideman et al., 2018). Notably, PDZD8 was recently shown to reside as well in MCSs between the ER and late endosomes/lysosomes (LE/Lys), where it interacts with the ER transmembrane protein Protrudin and the LE/Lys RAB7 GTPase (Elbaz-Alon et al., 2020; Khan et al., 2021), indicating that the molecular determinants of different MCSs can be interconnected, and shared in response to specific stimuli.

Besides ERMES or MAMs, yeast and mammalian cells may harbor another complex involved in tethering ER and mitochondria, *i.e.*, the highly conserved ER membrane protein complex (EMC). EMC is a transmembrane-domain insertase for integral and tail-anchored proteins into the ER membrane (Bai et al., 2020). In yeast, the EMC proteins were shown to interact with the TOM subunit Tom5, thereby allowing transfer of PS into mitochondria to sustain the synthesis of PE (Lahiri et al., 2014). In mammalian cells, a specific study reported the EMC subunits EMC4 and EMC7 to interact with LE/Lys-localized RAB7, while another study demonstrated EMC1 and EMC2 to bind SLC25A46, a mitochondrial metabolite carrier family protein required for the maintenance of mitochondrial PA, PS and PE levels (Janer et al., 2016). Interestingly, the latter study also revealed an interaction between SLC25A46 and Mfn2. It still remains to be clarified whether EMC acts directly as a tether to mediate phospholipid transfer, or rather indirectly by facilitating membrane insertion of other tethering and lipid-handling proteins, which would be in agreement with a recently suggested role of EMC in cholesterol homeostasis and maturation of sterol-regulating enzymes (Volkmar et al., 2019; Volkmar and Christianson, 2020).

Taken together, the three types of ER-mitochondria tethering detailed above were each linked to the transfer and metabolism of lipid and ions in these two organelles, suggesting a central role in global cellular homeostasis.

### Contact Sites Between Mitochondria and Vacuole (Yeast)/Endolysosomes (Mammals)

Mitochondria also engage to form MCSs with the vacuole in yeast and LE/Lys in mammalian cells. In yeast, they are known as the “vacuole and mitochondria patches” (vCLAMPs) and were proposed to have a role in the transfer of ions and amino acids, as well as to offer a bypass for lipid transfer in cases where ERMES formation is compromised (Elbaz-Alon et al., 2014; Hönscher et al., 2014). Initially, vCLAMP tethering was shown to depend on Vam6 and the vacuolar Rab GTPase Ypt7, both of which are subunits of the homotypic fusion and protein sorting (HOPS) complex (Elbaz-Alon et al., 2014; Hönscher et al., 2014). Later studies identified Tom40 as an OMM-binding partner of Vam6 and demonstrated the existence of a second vCLAMP tether based on the recruitment of Vps13 and the OMM protein Mcp1 (John Peter et al., 2017; González Montoro et al., 2018). Notably, Vps13 is a conserved lipid transporter (Dziurdzik and Conibear, 2021) that also localizes to the vacuole and to its tripartite contact formed with the nucleus and the perinuclear ER, which is known as the nucleus-vacuole junction (NVJ) (Lang et al., 2015), once again demonstrating how different MCSs can be associated. In both vCLAMP and NVJ contacts, Vac8 also attracts the sterol transporter Lam6 and, similarly as described above for ERMES, this leads to an expansion of MCSs (Elbaz-Alon et al., 2015).

Whereas early studies on cholesterol and iron trafficking in mammalian cells already pointed to a physical interaction between mitochondria and the endolysosomal system (Sheftel et al., 2007; Charman et al., 2010; Kennedy et al., 2012; Das et al., 2016), the structural basis of these mito-LE/Lys contact sites is still poorly understood. The first tethering protein was only recently identified as being RAB7, the ortholog of yeast Ypt7, and demonstrated to be required for mitochondrial fission (Wong et al., 2018). Moreover, the lipid transporter VPS13A was reported to act as an additional mito-LE/Lys tether, similar to its yeast ortholog (Muñoz-Braceras et al., 2019) and the OMM protein GDAP1 was suggested to tether lysosomal LAMP1 (Cantarero et al., 2021). In addition, Mfn2 has been reported as a potential regulator of mito-LE/Lys contacts sites (Khalil et al., 2017). Functionally, contacts between mitochondria and early or late endosomes were shown to be important for the local translation of endosome-delivered mRNAs encoding mitochondrial proteins (Cioni et al., 2019; Müntjes et al., 2021; Schuhmacher et al., 2021), exchange of metabolites (Peng et al., 2020), and regulation of both mitochondrial dynamics and inter-mitochondrial contact sites (Wong et al., 2019).

This tight connection between the mitochondria and vacuolar/endolysosomal entities suggests a crucial interplay between these organelles to maintain cellular homeostasis, as well as a rapid way for dysfunctions to spread between these compartments, providing a possible explanation to their common joint disruption.

### Contact Sites Between the ER and Vacuole (Yeast)/Endolysosomes (Mammals)

The vacuoles/endolysosomes also share contact with the broadly distributed synthesis and storage entity that is the ER. In yeast, the vacuole was found, as mentioned above, to form tripartite contact sites with the nucleus and the peripheral ER at NVJs. NVJs were first characterized by a tether formed between the vacuolar membrane protein Vac8 and the nuclear envelope protein Nvj1 (Pan et al., 2000) and later shown to represent the site for LD biogenesis upon recruitment of the sorting nexin-like protein Mdm1 and the seipin Sei1/Flp1 (Fei et al., 2008; Grippa et al., 2015; Hariri et al., 2018). NVJ contacts are known to expand upon nutrient stress and besides the sterol transporter Lam6, they contain several lipid-binding proteins such as the SMP protein Nvj2, the Mdm1 paralog Nvj3, the oxysterol binding protein Osh1/Swh1 and its ER-receptor Scs2, and the lipid transporter Vps13, which for its recruitment depends on the sorting nexin Ypt35 (Levine and Munro, 2001; Toulmay and Prinz, 2012; Elbaz-Alon et al., 2015; Henne et al., 2015; Bean et al., 2018; Weber-Boyvat et al., 2021).

In mammalian cells, the first ER-LE/Lys contact site pair identified was the ER-localized protein VAPA interacting with the LE/Lys-localized cholesterol-binding protein ORP1L (Rocha et al., 2009). Other LE/Lys-localized proteins with similar lipid motifs, such as StAR-related lipid transfer domain-3 (STARD3) and VPS13C, were more recently also shown to engage in MCSs with the ER (Alpy et al., 2013; Kumar et al., 2018) through binding of ER-localized VAPs (Wilhelm et al., 2016; Leonzino et al., 2021). Moreover, Niemann-Pick type C protein 1 (NPC1), which mediates lysosomal cholesterol egress, is partly considered an ER-LE/Lys tether as it binds to ER proteins such as ORP5 (Du et al., 2011) and Gramd1b (Höglinger et al., 2019). Interestingly, STARD3 (Wilhelm et al., 2016) was shown to relocate from ER-LE/Lys to mito-LE/Lys contacts upon inhibition of NPC1 (Höglinger et al., 2019), pointing to an interplay between these contacts. As mutations in NPC1 lead to the neurodegenerative lysosomal storage disorder of Niemann-Pick type C (Vanier, 2010), this supports the idea that an impaired MCSs network is central to neuronal demise. Focusing on ER-LE/Lys contacts, disruptions therein may lead to unfunctional exchange within the two organelles. Indeed, as the primary site of lipid biogenesis, the ER can directly transfer lipids to LE/Lys through MCSs. For cholesterol specifically, this was shown to allow the activation of the lysosomal mTORC1 signaling pathway that is essential for cellular homeostasis (Lim et al., 2019). In addition, as the major Ca^2+^ store in the cell, the ER also ensures the refilling of lysosomal Ca^2+^ through their shared contact sites (Garrity et al., 2016). A proposed mechanism involves contacts formed within ER areas that are particularly rich in IP_3_ receptors, allowing for a low-affinity uptake system to take place in the lysosome (Atakpa et al., 2018). Reversely, lysosomal Ca^2+^ can be released to the ER for subsequent signal amplification and storage (Hooper and Patel, 2012; Burgoyne et al., 2015). This transfer was suggested to help maintain MCSs with the ER, and to be mediated by two-pore Ca^2+^ channels (TPC) (Kilpatrick et al., 2017). In particular, TPC1 was shown to localize to ER-LE/Lys MCSs and be required for their formation (Kilpatrick et al., 2017). Finally, ER-LE/Lys MCSs also play an important role in endosomal maturation. The majority of endosomes share contacts with the ER as they mature, with an increase from 50–80% in the case of early, immature endosomes to 99% in late, mature ones. These contacts are maintained during the maturation of endosomes along microtubules, a process that is primarily mediated by the formation of a complex between ER-localized Protrudin and VAPs proteins and LE/Lys-localized RAB7 and PI3P (Raiborg et al., 2015). As previously mentioned, PDZD8 may also take part in the interaction with this complex (Elbaz-Alon et al., 2020; Khan et al., 2021). In addition, the tubular ER protein Reticulon-3L (RTN3L) was more recently found to be enriched at MCSs with endosomes along their maturation. This was shown to occur upon recruitment by RAB9, which marks a transition stage between early and late endosomes, and to allow for both endosomal maturation and cargo sorting regulation (Wu and Voeltz, 2021).

As implied, vacuolar entities and endolysosomes thus also represent central players in the organellar interactome that balance many cellular pathways, not only through their connection with mitochondria but as well based on their own independent interactions.

### Contact Sites With Lipid Droplets

As both the mitochondrial and endolysosomal contacts involve lipid transfer, and as lipid dyshomeostasis is also a common hallmark of major NDs including PD and AD, we extend here our interest to contact sites involving LDs, as their biogenesis and function are also highly dependent on efficient inter-organellar communication (Renne and Hariri, 2021; Rakotonirina-Ricquebourg et al., 2022).

LDs are dynamic lipid-filled intracellular compartments bound by a lipid monolayer that arise from the outer ER lipid layer in response to various stimuli, including nutrient and oxidative stress. They play a key role in cellular lipid homeostasis and can act as lipid sinks that sequester excessive or toxic lipids, thereby preventing lipotoxic and oxidative cell damage, and providing feedstock for membrane biogenesis, lipid signaling and energy production (Petan et al., 2018). To support their complex and central role in lipid homeostasis, LDs form MCSs with various organelles, including the ER. This process is largely mediated by the ER protein Seipin (Sei1/Fld1 in yeast, BSCL2 in human) (Szymanski et al., 2007; Fei et al., 2008; Sui et al., 2018; Yan et al., 2018; Joshi et al., 2021), which mediates stable contacts with LDs through lipidic bridges (Jacquier et al., 2011). In addition, sorting nexin SNX14, just as its yeast ortholog Mdm1, is recruited to ER-LDs contact sites as an LD formation and tether protein (Datta et al., 2019). Of note, the mammalian Rab GTPase RAB18 can also establish LD contacts with the ER (Ozeki et al., 2005), for which it relies on the NRZ tethering complex and associated SNARE proteins and on the RAB18 binding partner DFCP1 (Xu et al., 2018; Li et al., 2019).

LDs are often found in close proximity to mitochondria, with which they form contacts as well. In yeast, such contacts were suggested to allow for protein transfer based on the presence of several enzymes involved in lipid metabolism (*i.e.*, Ayr1, Hfd1, Pgc1) at this interface (Schuldiner and Bohnert, 2017). In mammalian cells, this is mediated by perilipins (PLIN1 and PLIN5) interacting with the OMM protein Mfn2, and was shown to facilitate lipid trafficking from LDs to mitochondria (Wang et al., 2011; Nguyen et al., 2017; Benador et al., 2019). Such lipid trafficking is also supported by the lipid transfer protein VPS13D and the ESCRT protein Tsg101 (Wang et al., 2021).

Finally, LDs also interact with the vacuole in yeast, with LDs accumulating at the NVJ interface. This interaction involves the lipid phosphatase Pah1, suggesting a direct role in lipid storage (Barbosa et al., 2015). In mammalian cells, although SNX14 was shown to mediate a contact between LDs and LE/Lys, more research is needed to confirm whether contacts between LDs and endolysosomes are conserved, possibly to ensure lipid trafficking (Schuldiner and Bohnert, 2017).

Together, this points toward an important interplay between organelles mediated by contact sites. In particular, mitochondrial and endolysosomal pathways again seem highly connected, not only through their direct association, but as well independently through contacts formed specifically with the ER and LDs.

## Inter-Organellar Membrane Contact Sites in Neurodegeneration: Focus on PD and AD

Mitochondrial and endolysosomal demise are increasingly considered as main organellar hallmarks of cellular dyshomeostasis in two common NDs, *i.e.*, PD and AD. The different MCSs involving these compartments are likely to form an elaborate communication network to support cellular functions. One can thus assume that disruption at the level of any player in this conserved organization may compromise the entire system, with communicating pairs serving as relays between more distanced organelles. However, it remains unclear (i) which organelle is affected first, (ii) whether this differs in familial *versus* sporadic forms of PD and AD, and (iii) how this would then downstream impact other organelles. On one hand, the extensive organelle interactome may be-at least in part-redundant and represent a robust way to maintain cell homeostasis, while on the other hand, when a particular organelle becomes too dysfunctional, MCSs may provide a gateway to accelerate defects through other organelles.

Observations gathered in yeast indicate that the organelle interactome is indeed likely to respond as a network to threat exposure, with multiple reports highlighting the necessity of dynamic changes in MCSs upon cellular stress. In respiratory growth conditions, the number of vCLAMPs decreases (Hönscher et al., 2014), already indicating that these contacts are needed under specific circumstances. Exposure to ZnCl_2_ or starvation leads to a drop in survival rate and growth when cells express mutant forms of Vam6 that cannot support vCLAMP formation (González Montoro et al., 2018). Moreover, in the fungus *Candida albicans*, Vam6 is needed to protect against oxidative stress (Mao et al., 2021). Double mutants of Lam6 and ERMES subunits are synthetically sick, while this is not the case for double mutants of Lam6 and Vam6 (Elbaz-Alon et al., 2015), although the reason is still unclear. It has been put forward that ERMES is always present in logarithmically growing cells in sufficient numbers to promote cell growth, while vCLAMPs are barely observed in such conditions and thus not able to maintain cellular homeostasis if ERMES suddenly fails. However, this observation may also indicate that ERMES can be sufficient in physiological conditions, while vCLAMPs are probably-together with ERMES-required when cells are exposed to stress, as is also the case in a neurodegenerative context. It is likely that such protective mechanisms are conserved in mammalian cells. Indeed, upon cellular stress, mitochondria equally form contacts with RAB5-positive early, more immature, endosomes (Hsu et al., 2018). Furthermore, in mammals, MCSs may exert neuronal-specific functions, underlining a specific involvement in NDs. For example, ER tubules in contact with Lys promote lysosomal fission in the pre-axonal region, and subsequent kinesin-1-dependent Lys translocation into the axon (Özkan et al., 2021). *Vice versa*, VAPA-mediated ER-Lys contacts are necessary for ER elongation and network formation, whereby Lys reposition ER tubules to adapt to metabolic changes and to promote axon outgrowth (Lu et al., 2020).

Taken together, besides the extensive research already performed on MAMs in mammalian cells related to NDs, it appears crucial to also explore other organelle contacts in more detail in these disorders, as well as their interplay. Therefore, the next section will be focused on extending the involvement of MCSs in PD and AD, from MAMs to endolysosomal contacts, in order to gain a more extensive perspective and analyze the preferential root of cellular impairments associated with these neurological pathologies.

### Parkinson's Disease

Parkinson's disease (PD) is a common neurodegenerative disorder, affecting 9.4 million people worldwide (Maserejian et al., 2020). Patients display muscle rigidity, bradykinesia, and resting tremor; however, other motor and non-motor symptoms develop as the disease progresses (Poewe et al., 2017). PD is a multifactorial disorder, caused by a combination of age, genetic and environmental factors. Whereas the majority of patients develop a sporadic form of PD, typically above the age of 65, about 5–15% of the cases have a clear familial history and exhibit earlier onset. Genetic studies identified over 90 PD risk genes in sporadic cases and around 20 causal genes in familial forms, of which the majority have been implicated in mitochondrial and lysosomal pathways (Balestrino and Schapira, 2020).

At the cellular level, PD is hallmarked by α-synuclein (SNCA, PARK1/4) accumulation and toxicity, the appearance of Lewy bodies (LBs) and Lewy neurites (LNs); oxidative stress; Ca^2+^, metal and lipid dyshomeostasis; and organelle dysfunction. The latter ranges from mitochondrial dysfunction to endolysosomal defects, as well as impaired functionality of the Golgi and ER stress (Bernal-Conde et al., 2019; Nguyen et al., 2019; Malpartida et al., 2021; Udayar et al., 2022; Zambrano et al., 2022). Interestingly, LBs and LNs also display lipid accumulation and a dense packaging of dysfunctional organelles, merged with membranous structures and filaments (Shahmoradian et al., 2019).

Several PD-associated mutations are found in lysosomal proteins or proteins involved with lysosomal functionality. Loss-of-function (LOF) mutations in the P5B-type ATPase and lysosomal polyamine exporter **ATP13A2 (PARK9)** are causative for a plethora of NDs, including PD (van Veen et al., 2014), and result in lysosomal polyamine accumulation and subsequent rupture (van Veen et al., 2020). Dysfunction of **Glucocerebrosidase 1 (GCase)**, a resident of the lysosomal lumen responsible for degradation of glucosylceramide and glucosylsphingosine, forms a risk factor for PD development (Sidransky et al., 2009; Ryan et al., 2019; Avenali et al., 2020). LOF mutations in the serine/threonine kinase **PINK1 (PARK6)** and E3 ubiquitin ligase **Parkin (PARK2)** represent the most common cause of autosomal recessive familial early-onset PD (Truban et al., 2017). PINK1 and Parkin are important mediators of mitochondrial-lysosomal communication to ensure mitochondrial quality control. They enhance the formation of mitochondrial-derived vesicles to shuttle oxidized mitochondrial proteins to the lysosomes for breakdown (McLelland et al., 2014). Moreover, PINK1 and Parkin are key regulators of mitophagy, a process by which redundant or too damaged mitochondria are engulfed by autophagosomes that subsequently fuse with acidic lysosomes for degradation, and which is impaired in PD (Hou et al., 2020). Several gain-of-function (GOF) mutations in the ***Leucine-Rich Repeat*** ***Kinase 2 (LRRK2, PARK8)*** gene have been identified in sporadic and familial PD patients (Lesage et al., 2007), of which the G2019S mutant is best described. LRRK2 localizes to permeabilized lysosomes, where it recruits and phosphorylates RAB10 and RAB35, next resulting in the recruitment of JIP4 to stimulate lysosomal tubulation and vesicle formation, a process that is termed “lysosomal tubulation/sorting driven by LRRK2” (LYTL) (Bonet-Ponce et al., 2020). Interestingly, LYTL is upregulated in case of LRRK2 G2019S expression, although the specific role of LYTL in PD pathogenesis remains to be elucidated (Bonet-Ponce et al., 2020). **Vacuolar protein sorting 35 (VPS35, PARK17)** LOF is causative for late-onset autosomal dominant familial PD (Wang et al., 2016; Sassone et al., 2021). Like PINK1 and Parkin, VPS35 regulates mitochondrial-derived vesicles intended for lysosomal delivery. VPS35 thereby targets dynamin-related protein 1 (DRP1), a protein mediating mitochondrial fission, to the lysosomes for degradation, thus also modulating mitochondrial morphology (Wang et al., 2016). VPS35 represents a key component of the retromer complex, which is crucial for endosome-to-trans Golgi recycling of the mannose-6-P receptor that binds newly synthesized lysosomal hydrolases in the Golgi for lysosomal delivery (Cui et al., 2019). It is thus not surprising that VPS35 LOF or deficiency impairs lysosomal degradative capacity (Cui et al., 2019), which may contribute to decreased clearance of SNCA, as lysosomal **SNCA** accumulation is observed in a VPS35 knockdown *Drosophila* model (Miura et al., 2014). Moreover, LAMP2A, the receptor for chaperone-mediated autophagy, is reduced in VPS35 deficient dopaminergic mouse neurons, suggesting that impaired chaperone-mediated autophagy also contributes to SNCA accumulation (Tang et al., 2015). SNCA levels, multimerization, and aggregation need to be tightly controlled, as SNCA mutations–such as A30P and A53T–and multiplications of the *SNCA* gene, cause autosomal dominant familial PD, with the number of multiplications correlating to the disease severity (Stefanis, 2012; Ganguly et al., 2021). Moreover, SNCA aggregates form the main component of LBs (Stefanis, 2012). The endolysosomal system is important for SNCA, as it reaches the intracellular environment *via* endocytosis (Masaracchia et al., 2018), and SNCA pathogenic mutants are reported to exploit this system by sequestering LC3B monomers into microaggregates on the late endosomal membrane, thereby stimulating SNCA release in exosomes that then affect other neurons (Stykel et al., 2021). Moreover, overexpression of SNCA and SNCA aggregates hamper lysosomal homeostasis, as shown by impaired autophagy, decreased degradative capacity, and affected lysosomal hydrolase and vesicle trafficking (Mazzulli et al., 2016; Tang et al., 2021; Teixeira et al., 2021).

As already evident by the phenotypes discussed above, endolysosomal dysfunction in PD manifests at different levels (Vidyadhara et al., 2019), from disturbed endocytosis to autophagy pathways and cathepsin-mediated cell death (Hou et al., 2020; van Veen et al., 2020; Zou et al., 2021), which also affects other organelle types. Indeed, decreased degradative lysosomal capacity is linked to impaired mitophagy in PD (Liu et al., 2019a; Clark et al., 2021), resulting in the accumulation of damaged mitochondria. *Vice versa*, mitochondrial defects are known to induce endolysosomal adaptations, with an acute insult or chronic mitochondrial malfunction leading to elevated or decreased endolysosomal biogenesis, respectively (Fernández-Mosquera et al., 2017). Mitochondrial dysfunction, due to exposure to mitochondrial toxins or knockout of mitochondrial(-related) proteins such as apoptosis-inducing factor (AIF), PINK1, and Parkin, results in mitochondrial production of reactive oxygen species (ROS) that subsequently distorts lysosomal functionality and morphology (Demers-Lamarche et al., 2016).

However, restoring the function of one organelle type does not necessarily lead to improvements in other affected organelle types. Mitochondrial biogenesis was found to be transcriptionally repressed by upregulation of the transcription factors KLF2 and ETV1 in Niemann-Pick disease type C (Yambire et al., 2019), a severe neurodegenerative lysosomal storage disorder. Silencing KLF2 and ETV1 restored mitochondrial functioning, but was insufficient to rescue the lysosomal defects (Yambire et al., 2019). LOF mutations in the PD-linked protein ATP13A2 result in both lysosomal and mitochondrial dysfunctions (Dehay et al., 2012; Grünewald et al., 2012; Gusdon et al., 2012; Estrada-Cuzcano et al., 2017; van Veen et al., 2020; Vrijsen et al., 2020). ATP13A2-mediated lysosomal polyamine export and subsequent mitochondrial distribution counter mitochondrial ROS production in synergy with the intracellular polyamine-synthesis pathway (Vrijsen et al., 2020). However, when restoring lysosomal pH by feeding ATP13A2 deficient cells with acidic nanoparticles, the mitochondrial superoxide production persists upon exposure to the polyamine synthesis inhibitor difluoromethylornithine (DFMO) (Vrijsen et al., 2020).

Such observations suggest that the communication between these organelles is impaired in PD and possible other NDs, which includes-amongst others-physical organelle contacts. Several causal PD genes are involved in the formation or modulation of these inter-organelle contact sites, which will be outlined in detail in the next sections, where we will also highlight potential links with the broader organelle interactome.

### Alzheimer's Disease

Alzheimer's disease (AD) is the most common neurodegenerative pathology and is defined by the co-occurrence of extracellular accumulation of β-amyloid (Aβ) peptides in amyloid plaques and intraneuronal deposition of hyperphosphorylated tau in neurofibrillary tangles (Alzheimer, 1906). According to the amyloid cascade hypothesis, initially posited in the 1990s, neuronal death is the result of a pathogenic cascade of events that originates from excess Aβ deposition (Beyreuther and Masters, 1991; Hardy and Allsop, 1991; Selkoe, 1991; Hardy and Higgins, 1992; Hardy and Selkoe, 2002; Selkoe and Hardy, 2016). Whereas evidence struggles to bridge amyloid plaque load and cognitive decline (Price et al., 1991), increased intraneuronal toxic Aβ levels and endolysosomal abnormalities may be the potential missing link between these two events (Gouras et al., 2000, 2010; Peric and Annaert, 2015). Indeed, such a disruption of cellular homeostasis could explain the range of metabolic defects associated with AD, which are broadly distributed in terms of functional implications as they range from dyslipidemia (Di Paolo and Kim, 2011) and disruption of Ca^2+^ shuttling (Alzheimer's Association Calcium Hypothesis Workgroup Khachaturian, 2017) to impairments in glucose catabolism and mitochondrial energy production (Butterfield and Halliwell, 2019).

Aβ is generated through the sequential processing of the amyloid precursor protein (APP) by β-secretase and of its C-terminal fragment (CTF) by the γ-secretase complex. This complex is made of nicastrin (NCT), anterior pharynx defective-1 (APH-1), presenilin enhancer 2 (PEN-2) and presenilin (PSEN) (Edbauer et al., 2003; Sato et al., 2007; Escamilla-Ayala et al., 2020). Two homologs of PSENs, *i.e.*, PSEN1 and PSEN2, competitively enter the complex to act as its catalytic core, ensuring the proteolysis of APP-CTF as well as of >100 other substrates, including the Notch signaling protein (Jurisch-Yaksi et al., 2013; Güner and Lichtenthaler, 2020). PSEN/γ-secretase exerts in itself two sequential cleavage activities inside the membrane environment: (i) endoproteolysis at the ε-site, generating the APP intracellular domain (AICD) and retaining Aβ fragments of 48 or 49 amino acids, then (ii) carboxypeptidase processing at the γ-sites, liberating Aβ peptides ranging from 38 to 43 amino acids in the extracellular space or in the lumen of intracellular compartments (Takami et al., 2009; Bolduc et al., 2016). Herein, longer forms, *e.g.*, Aβ_42_, have a higher propensity to aggregate, accelerating their deposition in amyloid aggregates (Jarrett et al., 1993; Zhao et al., 2007).

The PSEN homolog in γ-secretase defines the intracellular localization of the entire complex (Sannerud et al., 2016). PSEN1/γ-secretase is majorly active at the plasma membrane and in sorting/recycling endosomes, and mainly contributes to an extracellular pool of Aβ_40_. PSEN2/γ-secretase is restricted to late endosomes and lysosomes, where it generates an intracellular pool of Aβ_42_. This pool is exacerbated upon familial AD (FAD) pathogenic mutations affecting PSEN2 (Sannerud et al., 2016). Further, FAD mutations in PSEN1 decrease γ-secretase processivity, shifting the balance to longer peptides (Szaruga et al., 2015, 2017). Interestingly, FAD-PSEN1 mutations that most strongly decrease processivity tend to translocate γ-secretase to LE/Lys, exacerbating the toxic pool of intracellular Aβ_42_ (Sannerud et al., 2016). This emphasizes the impact of PSEN/γ-secretase activity taking place in LE/Lys on the generation of aggregation-prone intracellular Aβ that can then toxically accumulate in these compartments and contribute to pathology onset. Indeed, neurons facing an increased intracellular Aβ burden display dysfunctions of the endolysosomal pathway at early preclinical stages in various AD models (Cataldo et al., 2000; Takahashi et al., 2004; Peric and Annaert, 2015). This correlates with the aberrant accumulation of intracellular Aβ_42_ (Gouras et al., 2000) as well as of APP-CTFs (Woodruff et al., 2016; Hung and Livesey, 2018), both of which are indications of impaired PSEN/γ-secretase-mediated cleavage. Further, AD transgenic neurons present an enlargement of intracellular multivesicular bodies in response to Aβ accumulation, suggesting that the endolysosomal system might be an underappreciated site of initiation of toxic Aβ aggregation (Willén et al., 2017).

In any case, endolysosomal and mitochondrial abnormalities could be herein as well reconciled by MCSs serving as a portal for the affections to spread throughout the neuron.

## MAMs in PD and AD

**Figure 2:**
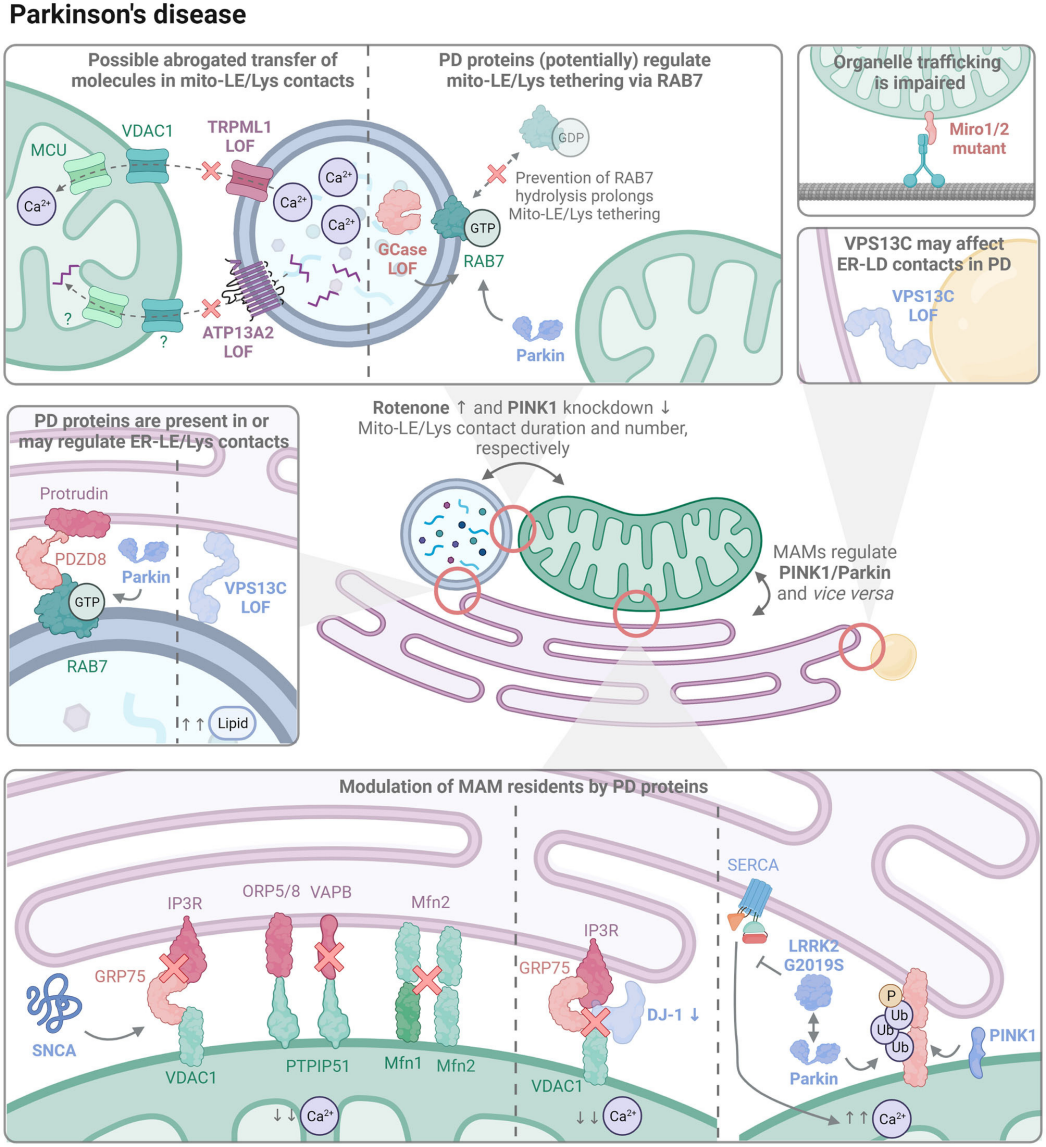
The organelle interactome in PD. PD-linked proteins (indicated in bold) are involved in the regulation of multiple contact sites, ranging from mitochondrial-late endo/lysosomal (mito-LE/Lys), endoplasmic reticulum-late endo/lysosomal (ER-LE/Lys), endoplasmic reticulum-lipid droplet (ER-LD), and mitochondrial-endoplasmic reticulum (MAMs) contacts. New hypothetical mediators and mechanisms are illustrated as well as established ones. LOF, loss-of-function.

**Figure 3:**
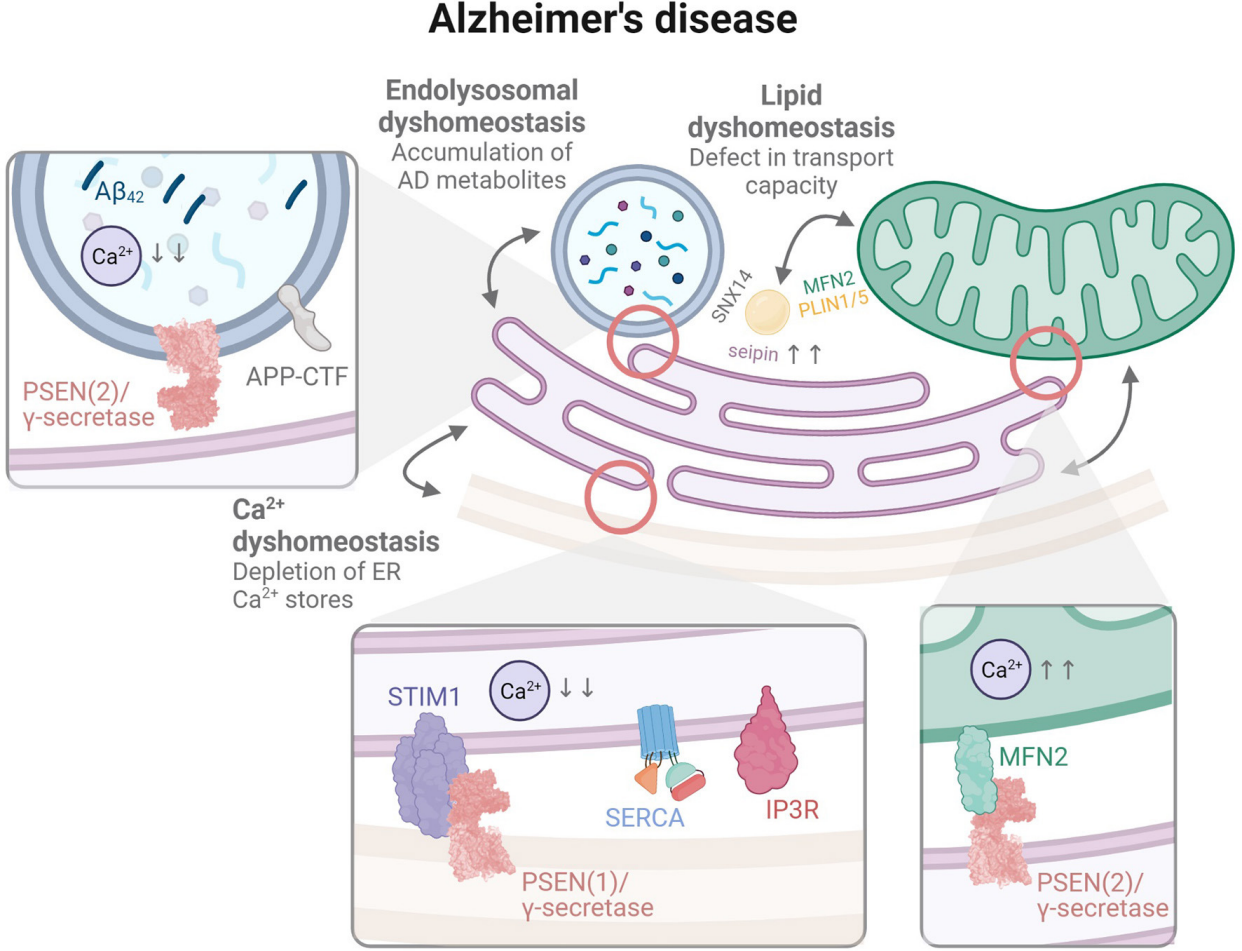
The organelle interactome in AD. Contact sites between the ER and mitochondria (MAM) were linked to a physical interaction between MFN2 and PSEN2/γ-secretase, while ER-LE/Lys contacts may represent a primary root for organellar disruption through the generation of aggregation-prone Aβ in LE/Lys by PSEN2/γ-secretase. The illustrated observation that lysosomal Ca^2+^ is disrupted in AD supports this hypothesis. Contacts between the ER and the plasma membrane are additionally represented in order to illustrate disruption of Ca^2+^ homeostasis involving the capacitative SOCE mechanism, with a suggested interaction between PSEN1/γ-secretase and STIM1.

Thus far, MAMs have received the most attention in the neurodegeneration field. However, as stated above, the sole impairments in these specific contacts are not sufficient to explain all cellular dysfunctions associated with NDs, in particular PD and AD. In addition, three or even more organelle types can come together in one contact (Valm et al., 2017). For example, mitochondrial fission is regulated by mito-LE/Lys contacts that are also positive for ER (Wong et al., 2018). Given the close interplay between MCSs, our aim here is to first provide a brief overview of MAM alterations in PD and AD [which has been reviewed elsewhere, see (Gómez-Suaga et al., 2018; Raeisossadati and Ferrari, 2020; Leal and Martins, 2021; Lim et al., 2021; Ray et al., 2021; Ziegler et al., 2021)], to then focus on the more poorly characterized MCSs (for a graphical abstract, see Figures 2, 3, respectively).

### Parkinson's Disease

Whereas mitochondrial dysfunction is one of the major contributors in the pathogenesis of PD, also ER stress has been implicated (Rodríguez-Arribas et al., 2017). Some PD-related proteins localize at mitochondria or MAMs and have been shown to participate in ER-mitochondria signaling. Among the PD-linked genes, PINK1, Parkin, LRRK2, SNCA, DJ-1, and Miro1/2 are directly linked to MAM homeostasis.

**PINK1** and **Parkin** are present in MAMs, especially in conditions of mitochondrial stress (Gelmetti et al., 2017). MAMs may function as initiation sites for autophagosome formation, and PINK1–but not Parkin–recruits the autophagy-mediator Beclin 1 to the ER-mitochondria interface to aid in this goal (Gelmetti et al., 2017). PINK1 and Parkin LOF mutations either enhance (Celardo et al., 2016; Gautier et al., 2016) or decrease (Basso et al., 2018) the number and function of MAMs. In both cases, mitofusin also seems to play a role. On the one hand, mitofusin knockdown prevented the increased MAM tethering in PINK1/Parkin LOF cells (Celardo et al., 2016), although this may also be due to the fact that mitofusin forms a tether itself. Moreover, PINK1/Parkin phospho-ubiquitinate Mfn2, triggering Mfn2 mitochondrial extraction by p97, which subsequently increases the distance between mitochondrial and ER membranes (McLelland et al., 2018). On the other hand, Parkin-dependent ubiquitination on K416 of Mfn2 seems necessary for MAM formation, with Parkin LOF leading to fewer MAMs (Basso et al., 2018). One possible explanation for the discrepancy in these results may be the position and type of ubiquitination on Mfn2. Upon activation by PINK1, Parkin is known to promote different types of ubiquitin chain linkages (Ordureau et al., 2014), which is exemplified by the mono-*versus* polyubiquitination of VDAC1, a MAM tether protein, regulating apoptosis and mitophagy (Ham et al., 2020). Furthermore, Parkin mutations present different efficiencies in ubiquitin chain generation (Ordureau et al., 2014; Ham et al., 2020), but it remains to be established how this would affect MAM formation and function. MAMs also regulate PINK1 levels, and therefore possibly affect downstream PINK1/Parkin-dependent mitophagy. Normally, PINK1 localizes on the OMM, where it is cleaved by the inner mitochondrial membrane (IMM) protease PARL for subsequent degradation (Harper et al., 2018). If mitochondria are stressed, processing of PINK1 is hampered, resulting in PINK1 accumulation on the OMM, which triggers mitophagy. Recently, it was shown that cleaved PINK1 interacts with components of the ER-associated degradation (ERAD) machinery, leading to PINK1 ubiquitination and subsequent targeting to the proteasome (Guardia-Laguarta et al., 2019).

Contrasting observations have been reported regarding the effect of GOF mutant **LRRK2** G2019S on MAMs, with studies showing an increased (Lee et al., 2019) or decreased (Toyofuku et al., 2020) tethering. LRRK2 G2019S suppresses sarco/endoplasmic reticulum Ca^2+^ ATPase (SERCA), which pumps Ca^2+^ into the ER lumen, thus leading to depletion of ER Ca^2+^ levels. Paradoxically, this causes an enhanced MAM-mediated transfer of Ca^2+^ from the ER to the mitochondria, resulting in mitochondrial Ca^2+^ overload, increased ROS production, and mitochondrial fragmentation (Lee et al., 2019). However, LRRK2 G2019S also dissociates from E3 ubiquitin ligases, such as Parkin, so that PERK can phosphorylate and thereby activate them, which ultimately converges in ubiquitin-mediated degradation of MAM tethering proteins and thus less MAMs (Toyofuku et al., 2020). Additional factors remain unknown that would explain in which situation LRRK2 G2019S induces *versus* inhibits MAM formation.

**SNCA** localizes to MAMs, while mutant forms are less abundant and seem to prefer mitochondrial localization (Guardia-Laguarta et al., 2014). When overexpressed, wild-type and mutant SNCA bind and disrupt tethers: SNCA specifically hampers the IP_3_R/GRP75 interaction in the IP_3_R/GRP75/VDAC1 complex (Erustes et al., 2021), and binds VAPB in the PTPIP51/VAPB tether (Paillusson et al., 2017). Moreover, in SNCA A53T transgenic mice, Mfn1 and Mfn2 levels decrease in an age-dependent manner, possibly contributing to the reduced MAM tethering (Xie and Chung, 2012) and speculatively also leading to less mito-LE/Lys contacts, as this is observed upon Mfn2 knockdown in erythroid progenitors (Khalil et al., 2017). The SNCA-dependent MAM disruption impairs the Ca^2+^ transfer from ER to mitochondria, and leads to a decreased ATP production and mitochondrial fragmentation (Paillusson et al., 2017; Erustes et al., 2021). Importantly, the expression level of SNCA appears to be a key factor in the modulation of MAMs. When acutely increasing SNCA, a slight upregulation enhanced the mitochondrial Ca^2+^ load and thus possibly MAM formation, whereas a steep upregulation or knockdown of SNCA had the opposite effect (Calì et al., 2012).

**DJ-1 (PARK7)** LOF mutations lead to autosomal recessive familial PD (Repici and Giorgini, 2019). DJ-1 is a multifunctional protein, sensing oxidative stress, modulating transcription, and exerting protease and chaperone activities (Ariga et al., 2013). Recently, DJ-1 was identified as part of the IP_3_R/GRP75/VDAC1 tether in MAMs, where it helps to maintain these MCSs. Downregulation of DJ-1 or expression of the disease mutant DJ-1 L166P results in disruption of the tethering complex, leading to decreased MAM formation, reduced mitochondrial Ca^2+^ load, and decreased ATP production (Liu et al., 2019b).

Mutations in **Miro1/2** emerge as genetic risk factors for developing PD (Grossmann et al., 2020; Nguyen et al., 2021). Miro1/2 are regulated by PINK1 and Parkin, and function as mitochondrial Rho GTPases that connect with MICOS (Liu et al., 2012). They are crucial for mitochondrial dynamics, as they couple mitochondria to trafficking proteins (Birsa et al., 2013; Modi et al., 2019), and Miro1 deficient mouse neurons display failure of retrograde axonal trafficking of mitochondria (Nguyen et al., 2014). Mutant Miro1 decreases MAMs in patient-derived fibroblasts (T351A, T610A, R450C, R272Q Miro1) (Berenguer-Escuder et al., 2019; Grossmann et al., 2019), whereas the R272Q Miro1 mutant enhances MAM formation in human iPSC-derived neurons (Berenguer-Escuder et al., 2020), indicating a cell-dependent effect. In all cases, MAM function seemed impaired, as there was decreased mitochondrial Ca^2+^ buffering and diminished autophagosome formation. Interestingly, contacts among ER, Golgi, Lys, peroxisomes, mitochondria as well as LDs are shown to depend on an intact microtubule network (Valm et al., 2017), underlining the need of intracellular trafficking for the proper formation of the organelle interactome, and suggesting that Miro1 defects may affect multiple (mitochondrial) contacts besides MAMs. Moreover, Miro1 on the mitochondria and a peroxisome-localized Miro1 splice variant were shown to recruit the lipid transporter VPS13D, which binds ER-localized VAP (Guillén-Samander et al., 2021), thereby coupling the ER to both mitochondria and peroxisomes. VPS13D also maintains mitochondrial-LD interactions (Wang et al., 2021), adding to the possibility that multiple contacts may be altered in PD in case of Miro1 deficiency or dysfunction.

Taken together, MAM formation and functionality is reported to be either increased or decreased in PD models, depending on the cell type, mutation, and protein investigated. Despite these apparent discrepancies, impaired Ca^2+^ homeostasis and mitochondrial dysfunction have continuously been reported, pointing to a delicate equilibrium in MAM formation that needs to be maintained. Moreover, disease-associated genes may have an impact on multiple MCSs which may further explain the complexities of the observed MAM phenotypes.

### Alzheimer's Disease

A particular role for **PSENs** in mitochondrial dysfunctions, which appear relatively early in AD models (Hauptmann et al., 2009), was put forward based on their reported presence in MAMs. Area-Gomez, Schon and colleagues first showed an enrichment of both PSENs in MAMs (Area-Gomez et al., 2009) isolated from whole mouse brains (Lewin et al., 2002). They described the presence of a full active γ-secretase complex, as demonstrated by cleavage of exogenous substrates and endogenous liberation of AICD. The same research group then addressed the functional implication of PSENs at MAMs in PSEN deficient mouse embryonic fibroblasts (MEF) and fibroblasts isolated from AD patients, including MAM (i) formation, *i.e.*, ER-mitochondria connectivity and (ii) function, *i.e.*, MAM-dependent lipid metabolism, as well as (iii) ER-mitochondria communication (Area-Gomez et al., 2012). Consequently, confocal microscopy revealed an increased ER-mitochondria apposition, *aka* MAMs, in both PSEN-deficient MEF and AD fibroblasts. This correlated with elongated ER-mitochondria contacts as observed by electron microscopy (EM). Evaluation of MAM function and ER-mitochondria communication further demonstrated an upregulation of the conversion of free cholesterol to cholesteryl esters and the trafficking and synthesis of phospholipids in both cell types. Together, these observations led the team to propose that the cellular impairments accompanying AD pathogenesis may originate from an upregulated MAM function at the interface between ER and mitochondria, and a prolonged cross-talk between these two organelles.

More recently, **PSEN2** was also implicated in modulating the coupling between the ER and mitochondria through a physical interaction with **Mfn2**, thereby reversing its negative regulation of MAM formation in MEF cells (Filadi et al., 2016). The PSEN2-Mfn2 interplay appeared to be favored in both transgenic mice and human fibroblasts bearing FAD-PSEN2 mutations, linking again upregulated MAMs and AD pathogenesis. In agreement, PSEN2-deficient MEF were shown to display an impairment in mitochondrial homeostasis. Biochemical analyses combined with EM and Seahorse^TM^ monitoring demonstrated that PSEN2 deficiency reduced the expression levels of mitochondrial oxidative phosphorylation (OXPHOS) complex subunits, the number of mitochondrial cristae where OXPHOS takes place, as well as oxygen consumption (Contino et al., 2017). These impairments were reversed upon PSEN2 re-expression, underscoring a specific implication for PSEN2 in the maintenance of mitochondrial morphology and homeostasis. However, and surprisingly, none of these disruptions could be recapitulated in primary neuronal nor astrocytic cells isolated from PSEN2-deficient transgenic mice (Contino et al., 2021). Moreover, the idea that upregulated MAMs could participate in AD pathogenesis was recently challenged. In a *Drosophila* model of AD, forced interactions between ER and mitochondria using an engineered linker increased locomotor activity, reversing the characteristic motor impairment of these flies, and extended their lifespan (Garrido-Maraver et al., 2020).

Although some studies have suggested the presence of APP-CTF and the generation of Aβ in MAMs (Schreiner et al., 2015; Pera et al., 2017; Leal et al., 2020), the rationale for this process contradicts what is currently known on the location of γ-secretase. Firstly, the dual processing of APP occurs essentially in post-Golgi compartments where also mature and active secretases reside (Sannerud and Annaert, 2009; Sannerud et al., 2011, 2016). Moreover, γ-secretase assembly was recently shown to be initiated in the ER through the formation of two heterodimers–NCT-APH1 and PSEN-PEN2 –, that only assemble into full complexes after ER-exit, and most likely in the intermediate compartment and/or cis-Golgi (Wouters et al., 2021). It is thus very unlikely that the PSEN/γ-secretase complex would exert cleavage activities on APP-related substrates in the ER or its contact sites prior to reaching the plasma membrane. Furthermore, it is not clear how and why these proteins would reach intracellular compartments such as mitochondria. Thus, there are still some discrepancies in the relation between MAM formation and functionality and the development of AD cellular pathology.

## Beyond MAMs: Other Contacts in PD and AD

The conflicting observations discussed above add to the idea that MAM dysfunction might not solely contribute to the start of cellular impairments in PD and AD. As mitochondria and ER both communicate with other organelles through MCSs, the observed defects might result from impairments in multiple subcellular compartments rapidly spreading to the rest of the neuron. MCSs may represent a gateway to propagate multi-organellar dysfunctions, eventually leading to overall neuronal demise. Indeed, impaired Ca^2+^ homeostasis, mitochondrial dysfunction and ER stress are often paralleled by endolysosomal damage and lipid dyshomeostasis, possibly pointing to a disturbed interplay of contacts between these compartments.

## Mitochondria-Late Endosome/Lysosome Contacts

### Parkinson's Disease

Genetic and functional evidence indicate that the crosstalk between lysosomes and mitochondria is critically disturbed in PD, ranging from mitophagy and mitochondrial-derived vesicles to contact sites. While this was already the topic of other excellent reviews (Bai et al., 2020; Deus et al., 2020; Ray et al., 2021; Cisneros et al., 2022), we here focus on mito-LE/Lys MCSs in light of new recent findings and possible connections to other contact sites.

Exposure of cells to the pesticide **rotenone**, a mitochondrial respiratory complex I inhibitor and environmental risk factor for PD (Tanner et al., 2011), prolongs the duration of mito-LE/Lys contacts (Wong et al., 2019). The longer tethering may immobilize organelles to save energy, as previously proposed for inter-mitochondrial contacts (Wong et al., 2019), and/or may promote the exchange of metabolites to stimulate organelle recovery. The mitochondrial uncoupler CCCP also increases mito-LE/Lys contact duration, which is prevented by **PINK1** knockdown (Rabas et al., 2021). Moreover, knockdown of PINK1 results in a diminished number of these contacts under basal conditions as well as upon CCCP treatment (Rabas et al., 2021), suggesting that PINK1 regulates mito-LE/Lys contacts, particularly in the case of mitochondrial stress.

The most common genetic risk factor of sporadic PD (Sidransky et al., 2009; Ryan et al., 2019; Avenali et al., 2020), ***GBA1***, also affects mito-LE/Lys contacts. *GBA1* encodes the lysosomal enzyme GCase, which is responsible for the breakdown of the lipids glucosylceramide and glucosylsphingosine. Defective untethering of mito-LE/Lys contacts has been observed in iPSC-derived dopaminergic neurons of PD patients with a *GBA1* LOF mutation (Kim et al., 2021). Interestingly, pharmacological stimulation of GCase activity in patient-derived neurons rescues the phenotype, while inhibition induces a prolonged mito-LE/Lys tethering in control neurons, indicating that the activity of GCase is crucial in the modulation of these contact sites (Kim et al., 2021). Such hampered untethering is caused by significant proteasomal degradation of the TBC1 Domain Family Member 15 (TBC1D15) (Kim et al., 2021), as mito-LE/Lys untethering is driven by TBC1D15-mediated RAB7 hydrolysis (Wong et al., 2018). Moreover, a disturbed RAB7 hydrolysis is associated with hampered inter-mitochondrial contact untethering and impaired mitochondrial fission (Wong et al., 2019), but it remains unclear whether this is also the case in *GBA1* mutant cells. Besides executing mitophagy and its involvement in MAMs, the PD-associated gene **Parkin** also regulates RAB7 activity (Lee et al., 2016) and may therefore modulate mito-LE/Lys contacts. GCase inhibition is further shown to worsen SNCA pathology (Henderson et al., 2020), possibly indirectly affecting MAMs, since SNCA disrupts these tethers, as discussed above. Conversely, the addition of pre-formed SNCA fibrils to the medium of primary hippocampal neurons reduces GCase activity (Henderson et al., 2020), thereby possibly prolonging mito/LE-Lys contacts.

The **Transient Receptor Potential Mucolipin 1 (TRPML1)** cation-permeable channel not only functions as an oxidative stress sensor, but also regulates Ca^2+^ and iron homeostasis. TRPML1 LOF mutations cause the lysosomal storage disorder mucolipidosis type IV (MLIV), leading to lysosomal and mitochondrial defects. TRPML1 has also been implicated in various neurological diseases, and because of the positive impact of TRPML1 on lysosomal biogenesis and autophagy (Scotto Rosato et al., 2019; Santoni et al., 2020), TRPML1 agonists have been considered for the treatment of PD (Poewe et al., 2017; Santoni et al., 2020). Importantly, TRPML1 operates in mito-LE/Lys MCSs to deliver lysosomal Ca^2+^ to mitochondria *via* the OMM and IMM proteins VDAC1 and the mitochondrial Ca^2+^ uniporter (MCU), respectively (Peng et al., 2020). TRPML1 determines mito-LE/Lys contact tethering dynamics and modulates mitochondrial Ca^2+^ levels (Peng et al., 2020), representing a novel mechanism of intracellular Ca^2+^ regulation. Interestingly, lysosomes depend on the ER for their Ca^2+^ supply (Garrity et al., 2016; Raffaello et al., 2016; Yang et al., 2018), and TRPML1 colocalizes with the ER Ca^2+^ sensor STIM1 (Tedeschi et al., 2022), suggesting that TRPML1 may be involved in ER-Lys communication as well. We can therefore envisage a scenario where ER stress may lead to deprivation of lysosomal Ca^2+^, thereby also depleting mitochondria from Ca^2+^ through TRPML1, resulting in a cascade that gradually affects multiple organelle types. Iron is known to be delivered to mitochondria in kiss-and-run contacts between endosomes and mitochondria (Das et al., 2016; Hamdi et al., 2016), while iron homeostasis is disturbed in PD (Ma et al., 2021). As TRPML1 contributes to the maintenance of physiological iron levels, its position at mito-LE/Lys contact sites may be beneficial in exerting this particular function, which remains to be investigated.

**DRP1** is a large GTPase regulating multiple organelle dynamics. It is required for mitochondrial fission and peroxisomal division (Smirnova et al., 2001; Imoto et al., 2020), and it induces the formation of ER tubules that wrap around mitochondria at the sites of mitochondrial fission (Friedman et al., 2011; Adachi et al., 2020). Rodent PD models show either increased (Filichia et al., 2016; Zhang et al., 2020) or decreased mitochondrial DRP1 localization and fission (Portz and Lee, 2021), depending on whether the model was generated using toxins such as rotenone/MPTP or by overexpression of a SNCA mutant, respectively, and this suggests that the localization of DRP1 is altered in conditions of acute *versus* chronic stress. Interestingly, a brain-enriched DRP1 isoform has recently been identified, called DRP1_ABCD_ (Itoh et al., 2018), which can be found at the interface between mitochondria and LE/Lys, although it remains unknown whether it affects the function of these contacts. Moreover, this isoform also localizes to the plasma membrane and peroxisomes. Therefore, DRP1_ABCD_ warrants further investigation, as it possibly impacts on multiple organelle(s) (contacts).

**ATP13A2 (PARK9)** is a lysosomal polyamine exporter that is genetically implicated in a spectrum of related NDs, including PD (van Veen et al., 2014, 2020). While polyamines serve vital functions in the cell, ranging from translational control to anti-oxidative and anti-inflammatory effects, excessive polyamine levels are linked to neurotoxicity (Ha et al., 1998; Lagishetty and Naik, 2008; Pegg, 2013, 2016), underlining the need to closely balance polyamine availability. Indeed, loss of ATP13A2 leads to lysosomal accumulation of polyamines, which causes lysosomal dysfunction, rupture and eventually cell death (van Veen et al., 2020). Conversely, the reduced availability of polyamines in the mitochondria exacerbates mitochondrial oxidative stress (Vrijsen et al., 2020). The impaired polyamine transfer suggests that polyamines are possibly distributed from lysosomes to mitochondria through mito-LE/Lys contacts. Fostering this hypothesis, YPK9, the ATP13A2 yeast ortholog, is picked up as binding partner of the yeast tethering protein Vam6 and even proposed to be present in vCLAMPs (Elbaz-Alon et al., 2014).

In conclusion, the recently discovered mito-LE/Lys contacts are dysregulated in PD, but how this affects organellar ion, lipid or metabolite exchange and impacts on the formation and function of other MCSs remains to be further elucidated.

### Alzheimer's Disease

While mito-LE/Lys contacts remain largely unexplored in the context of AD, some interactors discussed above can also be linked to AD pathogenesis. For this reason, we include a discrete section here highlighting their potential interest for further investigation.

Firstly, **TRPML1** was reported to participate in AD-associated cellular defects *via* its role as an autophagy regulator (Curcio-Morelli et al., 2010). More specifically, TRPML1 was found to be downregulated in APP/PSEN1 transgenic mice and accompanied by a disruption in the autophagy-associated PPARγ/AMPK/mTOR signaling pathway (Zhang et al., 2017). To note, mTOR complex 1 (mTORC1) was also recently found to activate mitochondria through its nutrient sensing function, an inter-organellar communication pathway that appears inhibited by Aβ aggregates (Norambuena et al., 2018).

In addition, the mitochondrial fission protein **DRP1** was reported to interact with Aβ, causing an excessive fragmentation of mitochondria in disease conditions (Manczak et al., 2011).

Interestingly, in all cases, the observed organellar dysfunctions can be linked to other contacts as well, particularly the ER-LE/Lys contacts, which will be discussed below. Together, this supports the idea that the organellar interactome functions as a network to ensure cellular homeostasis.

## Endoplasmic Reticulum-Late Endosome/Lysosome Contacts

### Parkinson's Disease

LOF mutations in the lipid transport protein **VPS13C** result in autosomal recessive PD (Lesage et al., 2016; Darvish et al., 2018; Monfrini et al., 2022). More recently, mutations in VPS13C have also been linked to dementia with Lewy Bodies, underscoring common underlying molecular mechanisms in the spectrum of Lewy Body diseases (Smolders et al., 2021). Moreover, it emerges as an interesting target, as restoration of lipid alterations in different models (Fanning et al., 2019) and in various cell types, such as PD neurons, astrocytes, and microglia, has been proposed as a promising therapeutic approach (Brekk et al., 2020). VPS13C contains motifs enabling it to bind to the ER, LE/Lys, and LD, so that it is positioned at the interface of ER-LE/Lys and ER-LDs (Kumar et al., 2018). Loss of VPS13C results in an increased number of lysosomes and accumulation of di-22:6-bis (monoacylglycerol) phosphate (di-22:6-BMP), a lipid enriched in endolysosomes and a biomarker for lipid storage disorders and neurodegeneration (Showalter et al., 2020; Hancock-Cerutti et al., 2021). On top, VPS13C knockout cells display a leakage of mitochondrial DNA into the cytosol and a lysosomal inability to degrade the consequently activated stimulator of interferon genes (STING), together culminating in the initiation of a cGAS/STING-induced inflammatory pathway (Motwani et al., 2019; Hancock-Cerutti et al., 2021). How loss of VPS13C causes mitochondrial defects is yet unclear. It may be that (i) the lysosomal defects propagate further into mitochondrial dysfunction, thanks to–amongst others–impaired removal of damaged or aged mitochondria, (ii) VPS13C may have an additional role in mito-LE/Lys contacts, as the yeast VPS13C ortholog Vps13 can be found in vCLAMPs. However, in mammals, VPS13C was not found in mitochondrial contact sites (Kumar et al., 2018) and therefore, a possible regulatory effect of VPS13C on mito-LE/Lys contacts may be indirect; for example through one of its binding partners, like RAB7, which is also needed for proper VPS13C localization (Hancock-Cerutti et al., 2021). RAB7 mediates Mito-LE/Lys contacts (see above) and when active and GTP-bound, RAB7 also binds to the ER-LE/Lys resident protein PDZD8 (Elbaz-Alon et al., 2020). Therefore, it is tempting to speculate that RAB7 is a crucial mediator of ER-LE/Lys-mito tripartite MCSs, possibly affecting the communication among these organelles in PD. Interestingly, inhibition of RAB7 GTPase activity could rescue the aberrant lysosomal morphology in primary cultured fibroblasts of PD patients with a **LRRK2** G2019S mutation (Hockey et al., 2015). A similar effect was obtained when knocking down the TPC2 Ca^2+^ channel (Hockey et al., 2015), also resulting in a decreased percentage of ER-Lys contacts (Kilpatrick et al., 2017). These observations suggest that ER-Lys contacts are affected in case of LRRK2 GOF.

### Alzheimer's Disease

As a primary site of intracellular toxic Aβ accumulation, we argue that the LE/Lys compartments appear as a credible primary root for multi-organellar dysfunctions. As mentioned before, LE/Lys directly share contact sites with the ER as well as with mitochondria, possibly forming a tripartite complex (Elbaz-Alon et al., 2015; Murley and Nunnari, 2016). In accordance, endolysosomal impairments appear early in AD pathogenesis. These were formerly defined as originating from initial disruptions of (i) pH acidification or (ii) Ca^2+^ metabolism, of which the maintenance may be associated with contact sites. Whereas Lee, Nixon and colleagues have put forward a hypothesis according to which pH defects would initiate endolysosomal defects in the context of AD through a PSEN1-dependent recruitment of the lysosomal v-ATPase proton pump (Lee et al., 2015), other research groups could not confirm this (Coen et al., 2012; Zhang et al., 2012), and showed instead impaired LE/Lys Ca^2+^ as the triggering factor of endolysosomal demise (Coen et al., 2012; Zhang et al., 2012; Peric and Annaert, 2015). In both cases, however, evidence agrees that functional impairment of **PSENs** causes endolysosomal abnormalities in AD, as modeled either by PSEN deficiency or FAD-associated mutations. PSENs were as well suggested to act as Ca^2+^ leakage channels at the ER membrane (Tu et al., 2006; Nelson et al., 2007, 2011; Brunello et al., 2009) and/or to modulate the opening of other Ca^2+^ channels at this site such as the IP_3_ receptor (Stutzmann et al., 2004; Cheung et al., 2010) and SERCA pump (Fei et al., 2008), providing a hypothesis to explain how their impairment may disrupt Ca^2+^ metabolism. Alternatively, PSENs were also found to contribute to the refilling of ER Ca^2+^ stores *via* the capacitative mechanism of store-operated Ca^2+^entry (SOCE). SOCE occurs when STIM1 detects a decrease in ER Ca^2+^ and activates ORAI channels at the plasma membrane to replenish Ca^2+^ stores through ER contacts. SOCE was shown to be attenuated in cells and mouse models bearing FAD-associated PSEN mutations (Yoo et al., 2000; Herms et al., 2003), possibly due to a decrease of **STIM1** expression (Greotti et al., 2019) or to STIM1 being itself a substrate for PSEN/γ-secretase (Tong et al., 2016). However, such a role for PSENs in Ca^2+^ storage at the ER was challenged as conflicting observations were gathered regarding whether PSEN impairment induces an increase or decrease of Ca^2+^ levels in the ER (Zatti et al., 2006; Shilling et al., 2012). Again, this drives toward the hypothesis that the observed impairments might originate elsewhere than the ER, and in this case, potentially in LE/Lys.

Thus, the bridge between intracellular impairments and AD pathogenesis seems reconcilable through endolysosomal defects arising from the impairment of PSEN-mediated functions. It is an attractive idea that such endolysosomal dysfunction may impact on the communication to other organelles through contact sites, including those found at the ER and mitochondria, thereby eventually compromising the whole neuronal network and leading to degeneration.

## Lipid Droplet Contacts

### Parkinson's Disease

Up until now, no LD contact impairment has been described in PD, but the lipid transport and PD-linked protein **VPS13C** represents an interesting candidate, as it is a resident of ER-LD contacts and also plays a role in LD formation, similar to its yeast ortholog (Yeshaw et al., 2019). Moreover, overexpression of wild-type SNCA results in LD accumulation (Alza et al., 2021), whereas SNCA localizes to LDs upon exposure of cells to oleic acid (Cole et al., 2002). Interestingly, such relocalization is also observed for the A53T mutant, but not for the A30P mutant, although they both promoted lipolysis (Cole et al., 2002).

### Alzheimer's Disease

Since the initial descriptions of AD, lipid buildup in the form of LDs has been reported in AD brain tissue. Recent studies link several AD genetic risk factors, including the most common–*i.e.*, homozygosity for the ε4 allele of the lipid transporter ApoE–to LD-dependent lipid homeostasis and the associated protection to oxidative stress. According to the emerging concept, increased mitochondrial ROS production induces neuronal peroxidation and synthesis of lipids that are transported to astrocytes and either metabolized or sequestered in LDs in an ApoE isoform-dependent way, thereby attempting to delay neurotoxicity. ApoEε4 shows reduced lipid transport capacity and distorted intracellular lipid homeostasis, thus contributing to AD pathogenesis (Moulton et al., 2021; Qi et al., 2021; Sienski et al., 2021). Furthermore, **seipin**, a regulator of ER-LD contacts, is highly expressed in hippocampal neurons and astrocytes. Seipin depletion enhances Aβ neurotoxicity and induces neuroinflammation in animal models (Qian et al., 2016), suggesting a direct link between loss of ER-LD contacts and AD pathogenesis. Of note, such contacts are also supported by **SNX14**, a member of the sorting nexin proteins family, several of which were shown to regulate APP intracellular trafficking (Lee et al., 2008; Schöbel et al., 2008; Okada et al., 2010; Zhao et al., 2012; Muirhead and Dev, 2014; Wang et al., 2014).

Together, LDs, through their participation in inter-organellar contact sites, are emerging as important players in lipid homeostasis, which is disrupted in AD as well as several other neurodegenerative diseases. While some of the described LD-contact interactors may directly impact on the intracellular trafficking of APP and its fragments and, by extension, on Aβ generation, the precise role and mechanisms of LD-organellar interactions in AD remain largely to be explored.

## Discussion

Formation and function of organelle contacts are disturbed in PD and AD, although to a different extent in various models, which triggers new questions and hypotheses for future research.

Firstly, remarkable discrepancies arise from conflicting observations in PD and AD models. For several PD-associated genes, opposite phenotypes on contact sites have been reported, which may depend on the expression levels, the type of mutation and the cell type. Investigating how organelle contacts may be affected in sporadic PD, can provide key information regarding the most relevant phenotypes for further study. In AD, the observations that up- and down-regulation of contact sites could be beneficial *versus* detrimental calls for more extensive research to be performed. In addition, it would be interesting to determine how environmental insults and aging affects organelle contacts. This would not only apply to PD and AD but also to other NDs in which organelle contact sites were shown to be disturbed, such as Charcot-Marie-Tooth disease (CMT) (Bernard-Marissal et al., 2019; Wong et al., 2019; Cantarero et al., 2021) and amyotrophic lateral sclerosis (ALS) associated with frontotemporal dementia (FTD) (De Vos et al., 2012; Stoica et al., 2014; Gómez-Suaga et al., 2018; Lau et al., 2018). Indeed, as most NDs are jointly hallmarked by proteinaceous aggregates and impairments of several cellular pathways, overwhelming neurons and leading to their degeneration, the investigation of how different organelles may impact on others through contact sites would greatly help in identifying better targets to these–to date–incurable pathologies.

Further, current research mostly focuses on contact sites between different organelle types. However, inter-organelle contacts (*e.g.*, inter-mitochondrial contacts) may also play a role. In addition, the cross-talk between organelle contacts and other forms of organelle communication (*e.g.*, mitochondrial-derived vesicles, mitophagy, *etc*.) remains unstudied. One limiting factor is that, for many contact sites in mammalian cells, the identification of the tethers remains incomplete. The idea that master regulators in mammalian cells could coordinate multiple contact sites, similarly as Lam6 in yeast, is attractive to explain how different contact sites could be easily and commonly altered in disease. Importantly, such proteins may represent valuable therapeutic targets.

Considering that a prominent feature of organelle contact sites is the exchange of metabolites, mapping organelle composition *via* multi-omics approaches could serve to understand how subcellular metabolite levels are altered in disease models, and whether this can be altered or rescued by modulation of organelle contacts or transporters present herein. In addition, intracellular trafficking is impaired in neurodegeneration (Rajendran and Annaert, 2012; Hunn et al., 2015), hampering organelle transfer, possibly indirectly impacting on contact formation. As such, aiming to repair the organelle interactome at the cellular level could serve to restore a balanced trafficking of proteins, lipids and ions that are required for the maintenance of cellular homeostasis.

Finally, future research urges us to understand which organelles and contact sites are first affected in disease, and how this downstream impacts on the whole organelle interactome, leading to overall cellular demise.

## Data Availability Statement

The original contributions presented in the study are included in the article, further inquiries can be directed to the corresponding authors.

## Author Contributions

PA, JVS, JW, PV, and WA conceptualized the theme of the review and contributed to the writing. SV, CV, and MD contributed to the writing. SV and CV integrated the review and generated the figures and table. All authors contributed in proofreading the manuscript.

## Funding

PV is funded by the joint efforts of the Michael J. Fox Foundation for Parkinson's Research (MJFF) and the Aligning Science Across Parkinson's (ASAP) initiative. MJFF administers the grant [ASAP-000458] on behalf of ASAP and itself. The authors acknowledge the financial support of KU Leuven Consortium InterAction (C15/15/073, to JW, JVS, PA, WA, and PV; KA/20/085 to WA), VIB (to WA and PA), the Fonds Wetenschappelijk Onderzoek-FWO–Flanders (G0C3620N and G0C4220N to WA; G094219N to PV; 1S88419N to SV; G0C7222N to JW; G094922N to PA), and SAO-FRA (#2020/0030 to WA).

## Conflict of Interest

The authors declare that the research was conducted in the absence of any commercial or financial relationships that could be construed as a potential conflict of interest.

## Publisher's Note

All claims expressed in this article are solely those of the authors and do not necessarily represent those of their affiliated organizations, or those of the publisher, the editors and the reviewers. Any product that may be evaluated in this article, or claim that may be made by its manufacturer, is not guaranteed or endorsed by the publisher.
